# Expression and functional validation of heat-labile enterotoxin B (LTB) and cholera toxin B (CTB) subunits in transgenic rice (*Oryza sativa*)

**DOI:** 10.1186/s40064-015-0847-4

**Published:** 2015-03-28

**Authors:** Ho Seob Soh, Ha Young Chung, Hyun Ho Lee, Hemavathi Ajjappala, Kyoungok Jang, Jong-Hwa Park, Joon-Soo Sim, Gee Young Lee, Hyun Ju Lee, Young Hee Han, Jae Wook Lim, Inchan Choi, In Sik Chung, Bum-Soo Hahn

**Affiliations:** Division of Environmental Agricultural Research, Gyeonggido Agricultural Research & Extension Services, Hwaseong, 445-784 South Korea; Department of Agricultural Biotechnology, National Academy of Agricultural Science, Nongsaengmyeong-ro 370, Jeonju-si, Jeollabuk-do 560-550 South Korea; Department of Genetic Engineering and Graduate School of Biotechnology, Kyung Hee University, Yongin, 446-701 South Korea

**Keywords:** *Oryza sativa*, Enterotoxin, Transgenic plant, Oral vaccine, Immunogenicity

## Abstract

We expressed the heat-labile enterotoxin B (LTB) subunit from enterotoxigenic *Escherichia coli* and the cholera toxin B (CTB) subunit from *Vibrio cholerae* under the control of the rice (*Oryza sativa*) globulin (*Glb*) promoter. Binding of recombinant LTB and CTB proteins was confirmed based on G_M1_-ganglioside binding enzyme-linked immunosorbent assays (G_M1_-ELISA). Real-time PCR of three generations (T_3_, T_4_, and T_5_) in homozygous lines (LCI-11) showed single copies of *LTB, CTB, bar* and T*nos*. LTB and CTB proteins in rice transgenic lines were detected by Western blot analysis. Immunogenicity trials of rice-derived CTB and LTB antigens were evaluated through oral and intraperitoneal administration in mice, respectively. The results revealed that LTB- and CTB-specific IgG levels were enhanced in the sera of intraperitoneally immunized mice. Similarly, the toxin-neutralizing activity of CTB and LTB in serum of orally immunized mice was associated with elevated levels of both IgG and IgA. The results of the present study suggest that the combined expression of CTB and LTB proteins can be utilized to produce vaccines against enterotoxigenic strains of *Escherichia coli* and *Vibrio cholera*, for the prevention of diarrhea.

## Background

Enterotoxigenic *Escherichia coli* (ETEC) and *Vibrio cholerae* cause diarrhea by colonizing the small intestine and producing enterotoxins. The heat-labile enterotoxin B (LTB) produced by *E. coli* and the cholera toxin B subunit (CTB) produced by *Vibrio cholerae* are similar in structure, function and immunogenicity. LTB and CTB comprise a 27-kDa A subunit with ADP ribosyl transferase activity and a pentamer of 11.6-kDa B-binding subunits. The LTB and CTB subunits bind to G_M1_-ganglioside (galactosyl-N-acetylgalactosamyl-syalil-galactosylglucosylceramide) receptors on intestinal epithelial cells. Binding of LTB and CTB subunits allows a portion of the toxic A subunit to enter the cytosol, initiating a series of cellular events leading to diarrhea (Williams et al. 1999). CTB is applied as an oral vaccine against cholera, and immune responses against CTB are thought to prevent the toxic A subunit from entering host cells (Clemens et al. 1991). LTB forms a 60-kDa pentamer that facilitates binding to the G_M1_-ganglioside receptors on host epithelial cells, allowing the LTA (heat-labile enterotoxin A subunit) to endocytose and cause toxicity (Mason et al. 1998).

The production of recombinant proteins, such as biopharmaceuticals and vaccines, is commonly performed using bacterial, yeast and mammalian cell expression systems (Wurm 2004; Sue et al. 2005; Terpe 2006; Chen 2012; Mattanovich et al. 2012; Tingfeng et al. 2013). Recombinant proteins used in vaccines are glycosylated; bacteria do not glycosylate proteins, while yeasts may hyper-glycosylate proteins and show poor expression (Prokop et al. 1991; Archer 1994; Harashima 1994). The production of vaccines in microorganisms requires large-scale fermenters and stringent purification steps to obtain sufficient amounts of recombinant protein (Schonberger et al. 1991; Lebens et al. 1993). Similarly, mammalian cell cultures are expensive, hindering production of large quantities of vaccines. The development of various plant expression systems has enabled the expression of foreign antigens in plant tissue as edible vaccine vehicles (Haq et al. 1995; Du et al. 2005; El Adab et al. 2007; Ko et al. 2009; Sim et al. 2009; Unni and Soniya 2010; Cui et al. 2011; Guo et al. 2012). Plant-based edible vaccines offer advantages of longer shelf-life, stability at room temperature without loss of immunogenicity, and reduced production costs (Streatfield et al. 2001). Plant systems also ensure correct protein folding and eukaryotic post-translational modifications, with relatively high protein yield compared with other expression systems. Recently, enterotoxins have been expressed in several plant species. CTB has been expressed in a number of plant species, including potato (Arakawa et al. 1997), tomato (Jani et al. 2002), tobacco (Jani et al. 2004), carrot (Kim et al. 2009), rice (Oszvald et al. 2008; Kajiura et al. 2013), and maize (Karaman et al. 2012). Similarly, LTB was expressed in transgenic tobacco (Kang et al. 2003; Kim et al. 2011), rice callus (Kim et al. 2010) and carrot (Rosales-Mendoza et al. 2007, 2008), including the hairy roots of *Solanaceous* species (Guzman et al. 2011), and transgenic lettuce (Kim et al. 2007). However, the majority of plant expression systems pose challenges for vaccine production. For example, the production of vaccines in tobacco requires costly purification processes due to the presence of toxic alkaloid by-products (Ma et al. 2003).

The production of edible vaccines in seeds is attractive due to accumulation of storage proteins in relatively small volumes, and provision of stable dry environments that allow a high level of recombinant protein production. The advantages of rice include the high grain yield, ease of transformation, high stability, and ease of scale-up. Furthermore, rice is a self-pollinating plant with a low risk of unintended gene flow. Studies on rice as bioreactors have focused on utilizing its potential as an efficient oral vaccine delivery system (Yang et al. 2008). To date, several researchers have reported use of rice seed for the production of CTB (Nochi et al. 2007; Kajiura et al. 2013; Yuki et al. 2013).

Serum from CTB-immunized individuals does not neutralize LT as effectively as it neutralizes CT, and LTB and CTB show significant immunological cross-reactivity (Ahrén et al. 1993). Many studies suggested that the combined expression of CTB and LTB would be an excellent option to induce the individual’s immunization against ETEC and *V. cholera* because of the similar mechanism of action of CTB and LTB (Svennerholm et al. 1983; Svennerholm et al. 1986; Lebens et al. 1996). Despite the importance, there were no reports available on the combined expression of CTB and LTB proteins in rice. Hence, in the present study, CTB and LTB were produced together in rice grains using recombinant DNA technology. The *CTB* and *LTB* transgene expression level was monitored from third to fifth generations (T_3_-T_5_), and the *in vivo* immunogenicity of rice-derived CTB and LTB was evaluated in mice following oral and intraperitoneal administration. Our findings support the fact that the combined expression of CTB and LTB proteins can be utilized in producing vaccines against ETEC and *V. cholera* to treat diarrhea.

## Results

### Vector construction

**Figure 1:**
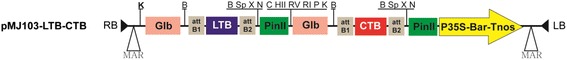
**Schematic representation of the binary vector containing LTB and CTB.** Glb, *Glb* promoter (0.9 kb); TpinII, *potato protease inhibitor* II terminator (1.0 kb); P35S, *CaMV 35S* promoter (0.84 kb); Bar, *bar* gene (0.55 kb); T*nos*, *nopaline synthase* terminator (0.25 kb); MAR, 5'-matrix attachment region of chicken lysozyme gene (1.3 kb).

The pMJ103 vector integrates the genes *LTB* and *CTB* in the inverted repeat regions through homologous recombination (Figure 1). The vector containing *LTB* and *CTB* genes is driven by the constitutive globulin promoter (*Glb*), along with potato protease inhibitor II terminator (*Pin* II). The expression vector contained the *LTB* and *CTB* genes, and the vector was labeled pMJ103-LTB-CTB (Figure 1). The *LTB* and *CTB* gene insertions were confirmed based on digestion of the vector with *Not*I. The digestion yielded about 3 kb of between the *CTB* inserts and *Pin* II (data not shown).

### Transformation and analysis of third-generation homozygous transgenic rice

**Figure 2:**
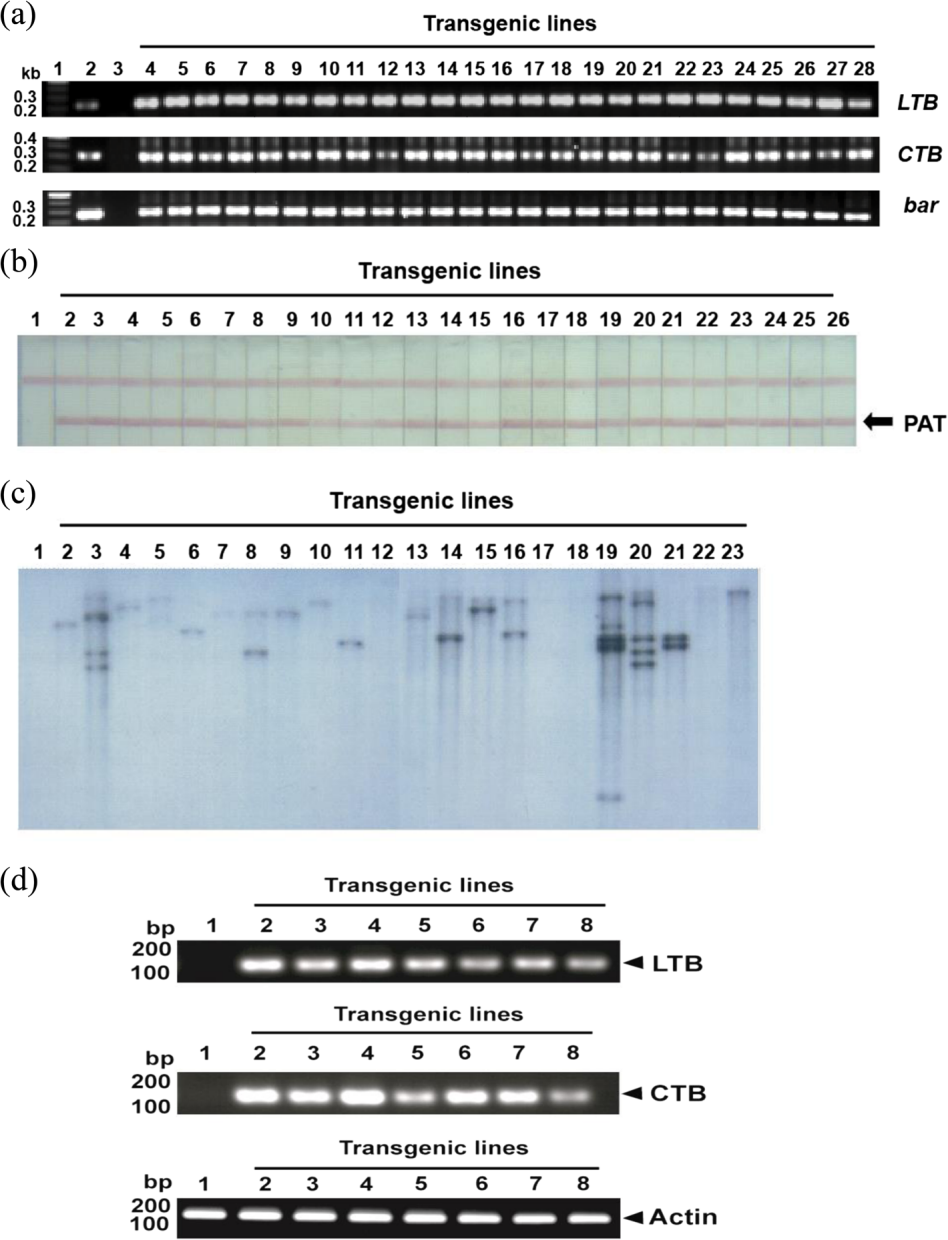
**Detection of** ***LTB, CTB*** **and** ***bar*** **genes and phosphoacetyl transferase (PAT) in putative transgenic plant leaf tissues. (a)** PCR amplification of *LTB* (217 bp), *CTB* (292 bp) and *bar* (302 bp) from transgenic rice genomic DNA. Lane 1 is the size marker. Lane 2 is the binary vector (pMJ103-LTB-CTB). Lane 3 is the wild-type. Lanes 4–28 are transgenic rice plants. **(b)** Lateral flow assay using the Trait LL lateral flow test kit (Strategic diagnostics). Lane 1 is the wild-type. Lanes 2–26 are transgenic rice plants. **(c)** Southern hybridization analysis of chromosomal DNA in transgenic rice. Genomic DNA from wild-type untransformed or independent transgenic rice T_0_ lines carrying the pMJ103-LTB-CTB construct was hybridized with a probe specific for the LTB coding region. A total of 10 μg of total leaf genomic DNA from transgenic rice plants was digested with *Xho*I, which cuts at a single site within the binary vector pMJ103-LTB-CTB. **(d)** LTB and CTB gene transcripts in transgenic plants were detected by RT-PCR. Plant RNA was isolated from selected transgenic plant rice seeds and RT-PCR was performed using a primer pair that specifically amplified 107- and 111-bp DNA fragments of the LTB and CTB genes (lanes 2 to 8). Lane 1 shows the RNA of non-transgenic plants used as a negative control.

Transformation in rice was performed using an *Agrobacterium*-mediated method. Putative transgenic calli were selected on MS media containing basta herbicide. The basta-selected calli regenerated into shoots and roots. A total of 25 putative transgenic rice plants was further screened for the presence of the *LTB*, *CTB*, and *bar* genes, and plants were designated T_0_. All 25 transgenic rice plants contained the *LTB*, *CTB*, and *bar* genes, based on PCR (Figure 2a). Insertion of the genes encoding the LTB and CTB genes was analyzed via PCR. PCR of transgenic rice plants revealed the expected 217- and 292-bp sequences, respectively (Figure 2a). Similarly, the transgenic plants were analyzed for PAT protein expression. Phosphoacetyl transferase (PAT) protein-specific band was detected in all 25 transgenic rice plants (Figure 2b). Transgenic plants regenerated from these events were subjected to Southern analysis using DNA obtained from young leaves of 22 lines, which revealed the specific integration of *LTB* (Figure 2c). Of the 22 plants, 3 were negative for copy number. Nine of twenty-two events involved a single copy number transgene insertion, and 9 of 22 involved multiple copy number transgene insertions.

RT-PCR analysis revealed LTB- and CTB-specific transcripts in total RNA extracted from transformed rice seeds (Figure. 2d). The amplification products corresponded to a specific major transcript of the expected size of the transgene (lanes 2 to 8). No bands were observed for PCR products of untransformed plants used as negative controls (lane 1). The internal control was actin-1, with a PCR product of 150 bp.

**Figure 3:**
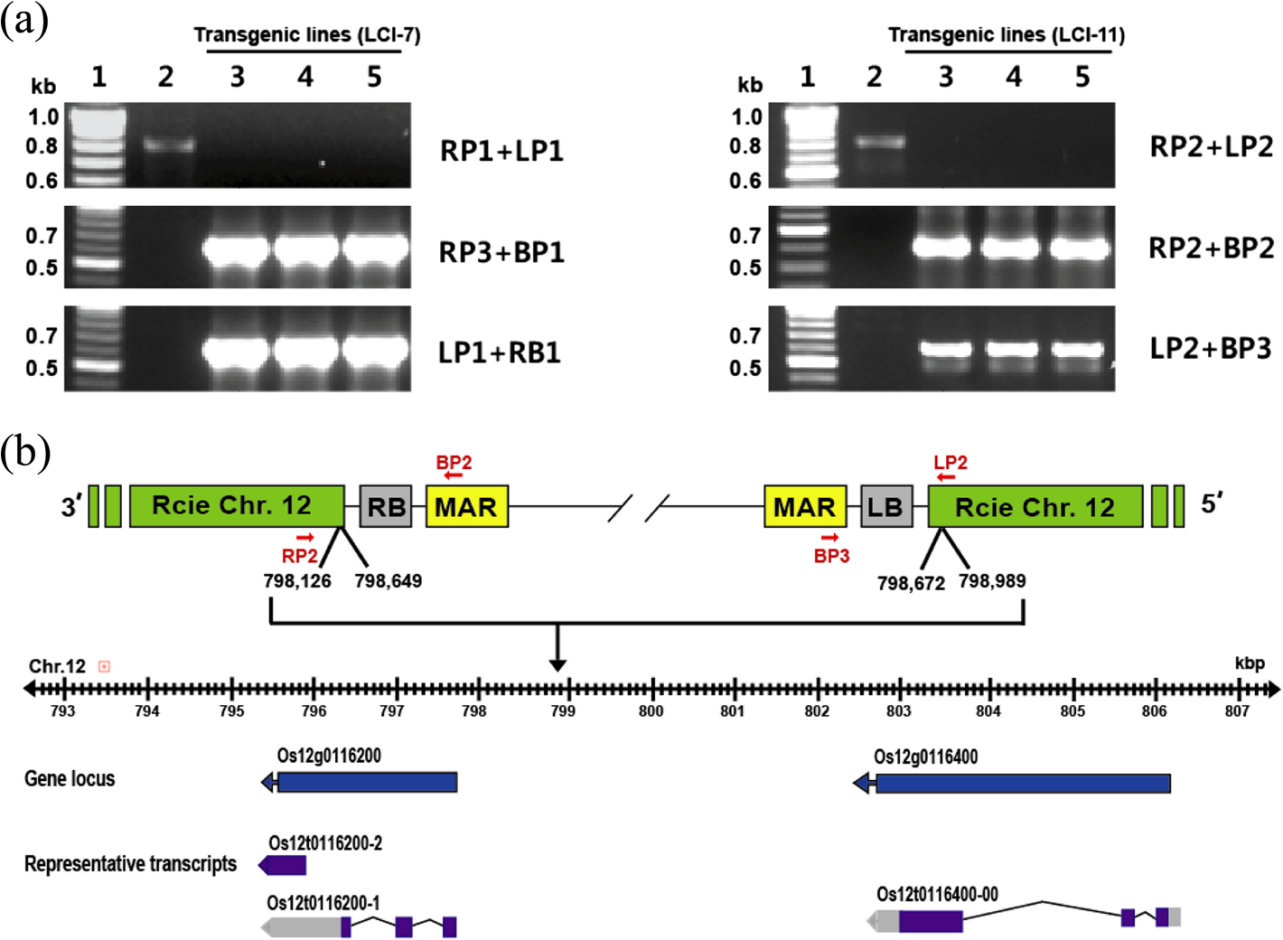
**Homozygosity of transgenic rice lines (LCI-7 and LCI-11) was analyzed by PCR using RP, LP, BP, and RB sequences. (a)** Three primer combinations were used in LCI-7 (left) and LCI-11 (right): the first comprised gene-specific right and left primers (RP1 + LP1 and RP2 + LP2); the second comprised a gene-specific right primer and T-DNA right border primer (RP3 + BP1 and RP2 + BP2); the third comprised a gene-specific left primer and a T-DNA left border primer (LP1 + RB1 and LP2 + BP3). Amplicon sizes are indicated on the right (in kb). **(b)** T-DNA insertion on chromosome 12 in the LCI-11 rice line. The chromosome regions in which T-DNA insertions were found ranged from 798,649 to 798,672 bp in length.

Analysis of left and right border chromosomal T-DNA localization in the transgenic lines showed the insertion of a T-DNA region. The localization of T-DNA ranged from chromosome number 1 to 12 with varying chromosomal sizes. The gene was found to be inserted in transgenic lines in the intron, exon, and intergenic regions, respectively (Table 1). Furthermore, the homozygous transgenic T_3_ lines (LCI-7 and LCI-11) analyzed by genomic DNA PCR revealed left border and right border sequences of *LTB* and *CTB* genes (Figure 3a,b). The results revealed a single band, indicative of homozygous lines (Figure 3a). The transgenic plants were grown to maturity to obtain the first- to fifth-generations (T_1–5_) seeds.

**Table 1:** **Characteristics of transgenic rice lines**

**Event ID**		**IPCR prod.**	**Rice Seq.**	**No Chr.**	**Chr. From** ^**#**^	**Chr. To** ^**#**^	**T-DNA end**	**Adaptor start**	**Copy number**	**Gene insert**
LCI-1	L*	302	302	3	35,147,954	35,147,653	−1	303	2	n.d
	R*	482	405	5	24,625,335	24,624,931	72	483		
LCI-3	R	542	536	8	26,724,580	26,724,045	−1	−1	1	intron
LCI-4	R	623	552	7	4,833,382	4,832,831	73	624	2	n.d
LCI-5	L	149	150	3	31,727,379	31,727,528	−1	150	1	exon
	R	448	343	3	31,727,348	31,727,006	67	449		
LCI-6	R	704	639	3	30,243,945	30,243,307	61	−1	1	intron
LCI-7	L	199	126	6	20,057,801	20,057,926	73	238	1	intergene
LCI-8	L	210	197	3	30,990,975	30,991,171	14	211	2	n.d
	R	347	275	9	17,404,938	17,405,212	72	348		
LCI-10	R	214	64	2	46,488	46,425			1	intergene
LCI-11	L	318	318	12	798,649	798,332	−1	319	1	intergene
	R	590	521	12	798,672	799,192	72	591		
LCI-13	L	395	395	9	16,474,041	16,474,435	−1	396	2	n.d
	R	211	184	5	24,829,622	24,829,805	25	212		
LCI-17	L	684	681	3	31,691,652	31,690,972	−1	−1	1	intron
	R	546	437	3	31,691,676	31,692,112	70	547		
LCI-18	R	217	146	1	32,792,438	32,792,293	72	218	1	intron

*L and R, left and right border sequences, respectively.

^#^Rice Annotation Project Database (RAP-DB) was used to retrieve rice annotation information.

N.d., not determined.

### Molecular analysis of third- to fifth- generations (T_3–5_) plants

**Figure 4:**
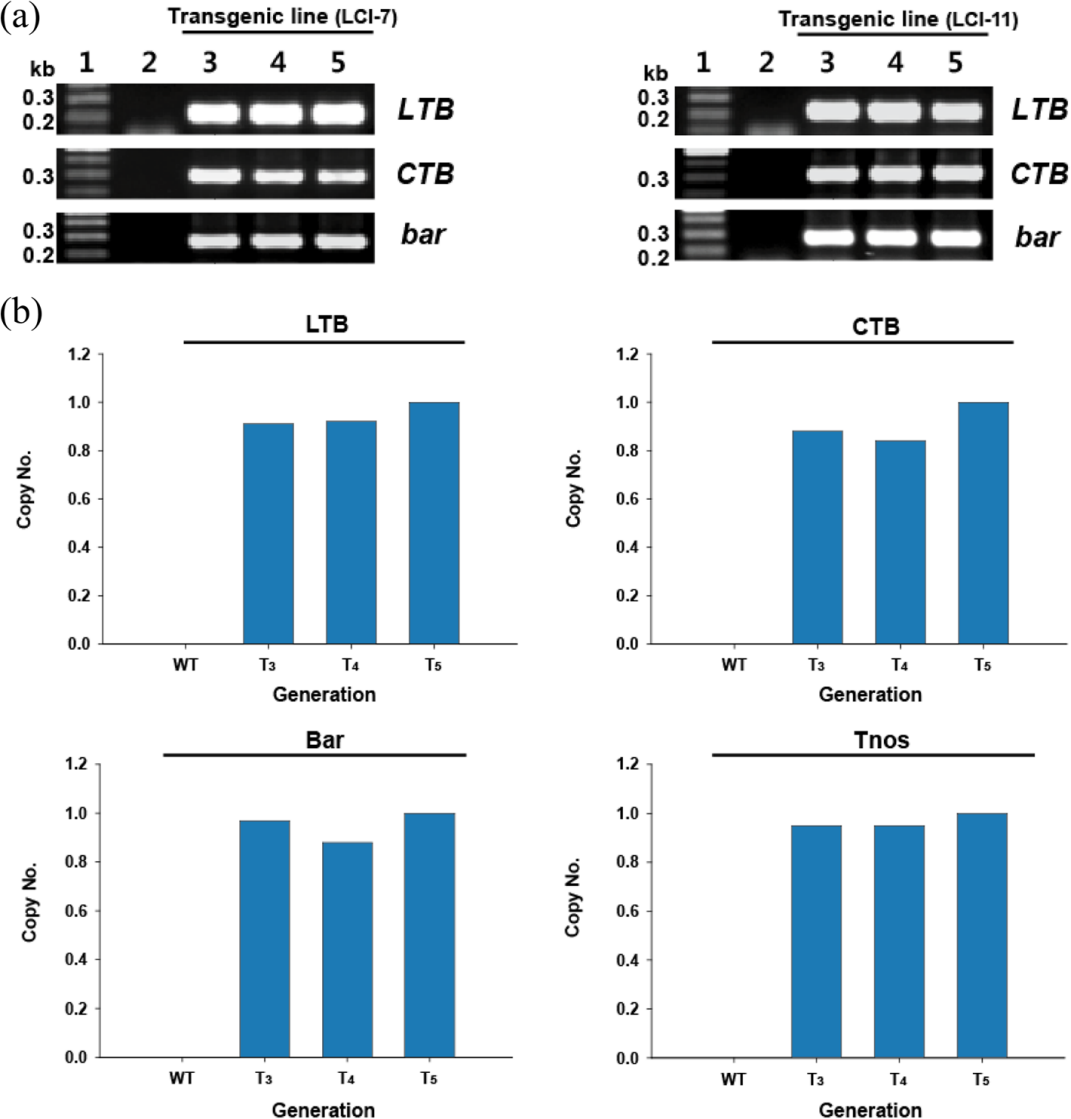
**Molecular analyses of transgenic plants by PCR and real-time PCR in the LCI-7 and LCI-11 lines (T**_**3**_ **, T**_**4**_ **and T**_**5**_ **generations). (a)** Genomic DNA isolated from T_3_ to T_5_ transgenic plants of LCI-7 (left) and LCI-11 (right) lines was used and the *bar*, *LTB*, and *CTB* genes were amplified by PCR. Lane 1: 1 kb marker: Lane 2: wild-type rice: Lane 3, 4 and 5: T_3–5_ transgenic rice. **(b)** The *LTB*, *CTB*, and *bar* gene, and T*nos*, copy numbers were analyzed in the T_3_, T_4_ and T_5_ generations of the LCI-11 rice line by real-time PCR.

Three transgenic (T_3_, T_4_ and T_5_ generations) lines obtained from T_2_ LCI-7 and LCI-11 transgenic plants were subjected to PCR to detect *LTB, CTB*, and *bar*; real-time PCR analysis was used to determine the copy number. PCR analysis revealed *LTB, CTB*, *bar*, and the T*nos* terminator (Figure 4a). *LTB*, *CTB*, *bar*, and the T*nos* terminator in the transgenic lines from three generations (T_3_, T_4_ and T_5_ generations) derived from the LCI-11 line were present as single copies, whereas no copies were detected in the control line (Figure 4b).

### G_M1_-ganglioside receptor binding assay of LTB and CTB proteins

**Figure 5:**
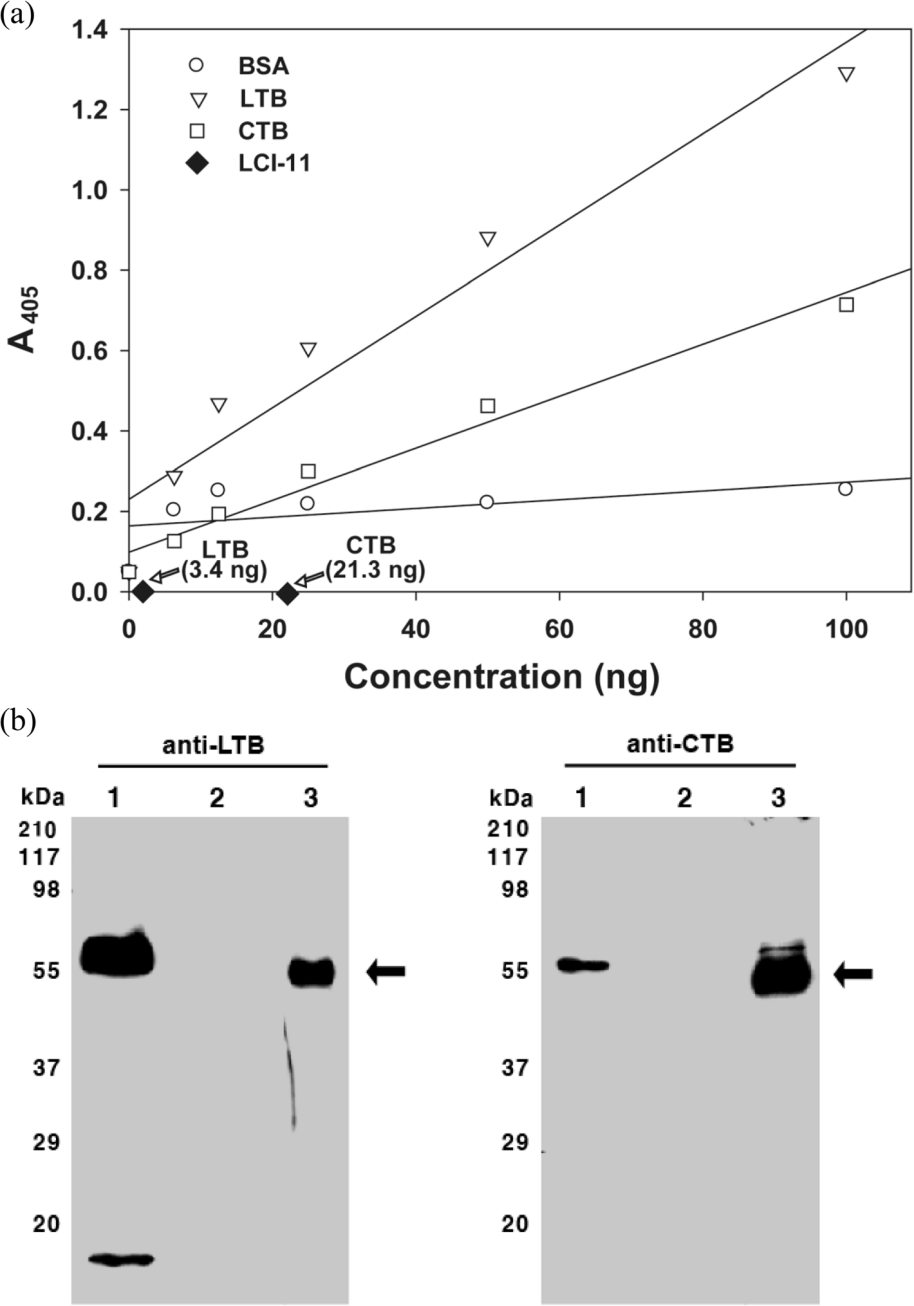
**Recombinant LTB and CTB protein expression by transgenic rice seeds. (a)** G_M1_-ganglioside receptor binding assay of LTB and CTB protein expression in transgenic rice. The relative amount of LTB and CTB proteins present in LCI-11 was estimated by standard curves of recombinant LTB and CTB proteins, respectively. Arrows indicate relative amount of LTB and CTB proteins in LCI-11. BSA, bovine serum albumin. **(b)** Western blot analysis of LTB and CTB expression by transgenic rice seeds using LTB- (left) and CTB- (right) specific antibodies, respectively. Lane 1: positive control. Lane 2: wild-type rice. Lane 3: transgenic rice. Arrows indicate LTB and CTB proteins.

Expression levels of LTB and CTB protein in seeds from the T_5_ transgenic line LCI-11 were determined based on ganglioside-dependent ELISA. The LTB protein accounted for 3.4 ng μg^−1^ of the total soluble protein and CTB accounted for 21.3 ng μg^−1^ of the total soluble protein compared to *E. coli*-synthesized LTB and CTB (Figure 5a). Because LTB and CTB show significant immunological cross-reactivity (data not shown), the concentration of LTB and CTB found in this study may vary from the actual concentration of LTB and CTB present in the transgenic rice seeds. The expression levels of LTB protein correlated with the single copy number obtained by Southern analysis. Furthermore, CTB and LTB protein expression was detected in the transgenic lines by Western blotting. Therefore, transgenic LCI-11 showed a band of 55 kDa, representing the LTB and CTB proteins (Figure 5b).

### LTB- and CTB-specific serum and feces IgG and IgA levels after mouse immunization

**Figure 6:**
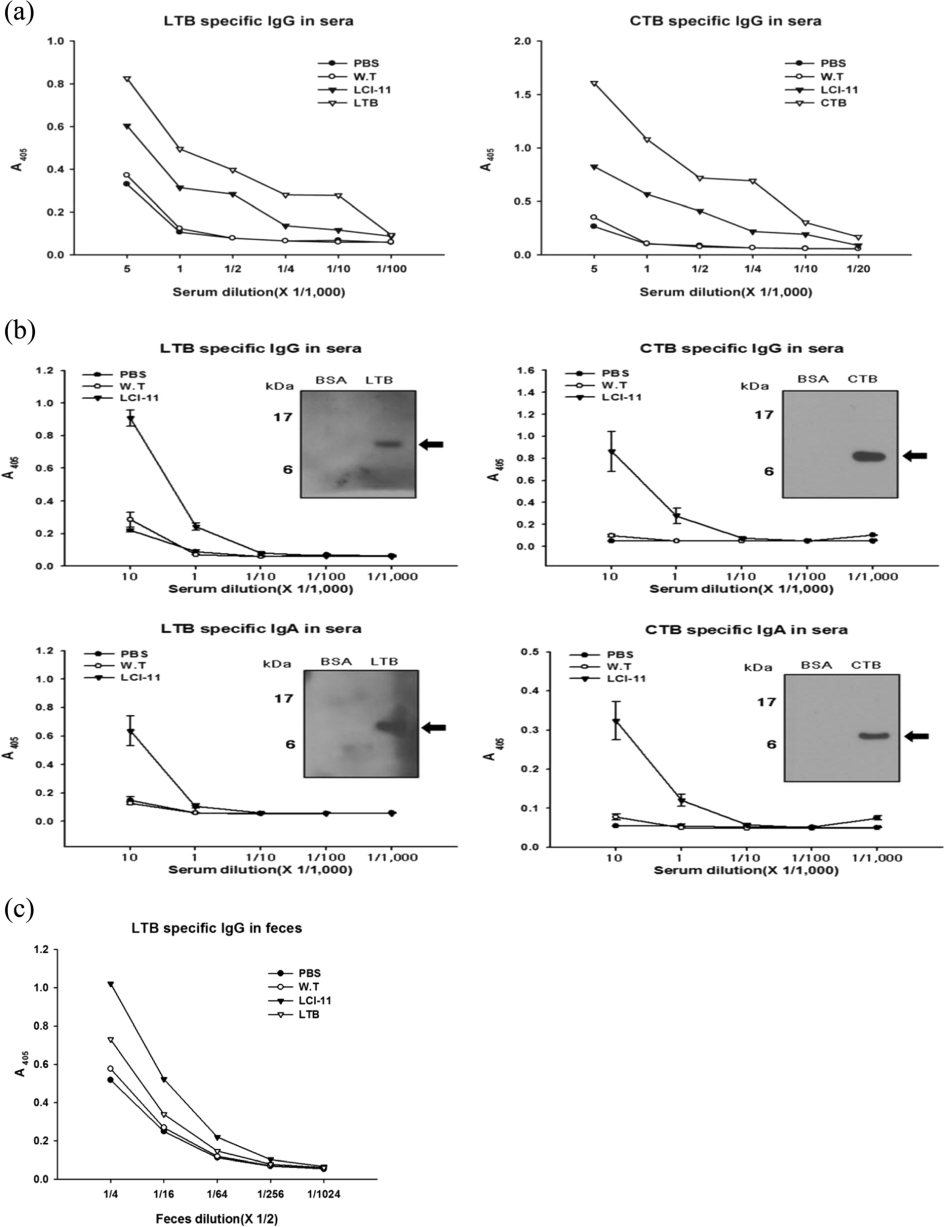
**Levels of LTB- and CTB-specific IgG and IgA in mouse serum and feces. (a)** CTB- and LTB-specific systemic IgG and mucosal IgA in mouse serum following intraperitoneal injection of wild-type (WT) and LCI-11 seeds and *Ed coli*-expressed LTB and CTB proteins. **(b)** LTB- and CTB-specific systemic IgG and mucosal IgA in mouse serum fed orally with wild-type and transgenic rice seeds. PBS was used as the negative control. Western blot (inset) detection of LTB- and CTB-specific IgG and IgA in mice immunized orally with LCI-11. First lane, BSA; second lane, LTB or CTB proteins, respectively. **(c)** LTB-specific mucosal IgA levels in mouse feces fed orally with recombinant LTB protein, wild-type and transgenic rice seeds. PBS was used as the negative control.

To determine whether the LTB and CTB proteins induced immune responses in mice, we administered LTB and/or CTB intraperitoneally (IP) and orally. For intraperitoneal immunization, 100 μg of LTB and CTB proteins were injected into mice. Purified, *E. coli*-expressed LTB and CTB proteins were used as positive controls. Serum samples collected from mice at various time points and the levels of anti-LTB and CTB IgG were determined up to a 10,000-fold dilution. Mice immunized with recombinant LTB and CTB proteins, and transgenic rice-produced LTB and CTB developed significantly higher levels of LTB- and CTB-specific serum IgG compared to the wild-type (Figure 6a). No serum LTB- or CTB-specific IgG was detected in mice immunized with a PBS control. Similarly, 100 mg of powdered samples in PBS from transgenic plants were fed orally to mice. Systemic LTB- and CTB-specific IgG and mucosal LTB- and CTB-specific IgA immune responses were induced in the sera collected from mice immunized orally with LCI-11 (Figure 6b). The level of IgG and IgA was similar in mice immunized with LTB (data not shown). Furthermore, western blotting detected LTB- or CTB-specific IgG and IgA antibodies in the serum of mice immunized with LCI-11 (Figure 6b). In addition, mice immunized with transgenic rice seeds or LTB protein developed significantly higher levels of LTB-specific IgA in feces compared with those immunized with wild-type rice seeds (Figure 6c).

## Discussion

Cholera and diarrheal diseases caused by enterotoxigenic *Escherichia coli* (ETEC) and *V. cholerae* are major global health problems. Cholera is the most severe ETEC infection, causing a large number of cases each year (Notaro and Kaper 1998). Effective vaccines could confer protection against these diseases by inducing an immune response and inhibiting enterotoxin activity. Immune-response-inducing toxins such as LTB and CTB are currently used as oral vaccines against diarrheal diseases. However, the production of these vaccines is expensive and requires stringent purification steps and delivery. Previously, several groups have demonstrated the production and oral immunogenicity of CTB and LTB in diverse transgenic plants (Jani et al. 2004; Rosales-Mendoza et al. 2008; Kim et al. 2009; Kajiura et al. 2013; Karaman et al. 2012; Yuki et al. 2013). Although several plant species are used for production of edible vaccines, seed crops—such as maize, wheat, or rice—are the most suitable. Among them, the use of transgenic rice for vaccine production offers several advantages over other plants. The benefits of producing edible vaccines in rice seeds include the production of large amounts of vaccine antigen for immunization, extended storage times at room temperature, and effective delivery of the vaccine to mucosal tissues.

Results from the present study indicate the potential for vaccine production in rice, an important cereal crop. In the current study, we focused on expressing CTB and LTB proteins in rice (*Oryza sativa.* L). A total of 25 putative transgenic rice plants showed the presence of *CTB*, *LTB* and *bar* genes, and plants were designated T_0_. The T_0_ plants harbored single and multiple copies of the LTB gene, as determined by Southern blotting. Expression levels of LTB and CTB proteins in grains were determined based on ganglioside-dependent ELISA. Levels of both proteins increased when compared to the BSA standard (Figure 5a). LTB and CTB from transgenic rice seeds accounted for up to 3.4 ng and 21.3 ng μg^−1^ per the total soluble protein, respectively. Enhanced CTB and protein levels were reported previously in cereal crops, at up to 0.05 μg in maize (Karaman et al. 2012) and 30 μg and 2.35 mg in rice (Dong et al. 1996; Kajiura et al. 2013). Similarly, LTB levels were reported to be 3.7% (Chikwamba et al. 2002) and 1.8% (Streatfield et al. 2002) in maize. Western blot analyses using an anti-CTB polyclonal antibody showed a molecular mass of 55 kDa. Previous studies reported a similar size, which was attributed to the pentameric structure (Nochi et al. 2007; Karaman et al. 2012). The CTBs expressed in rice were in their native pentameric form and were functionally active in terms of inducing antigen-specific immune responses.

Furthermore, intraperitoneal and oral immunization of mice with transgenic rice expressing LTB and CTB induced both systemic (IgG) and mucosal (IgA) immune responses (Figure 6a, b). Administration of 100-μg rice-expressed LTB and CTB administered IP was sufficient to induce LTB- and CTB-specific IgG production (Figure 6a). The levels of LTB- and CTB-specific IgG following IP administration in mice were similar. Furthermore, oral immunization of mice with LTB induced both systemic and mucosal IgA production. These findings are in agreement with previous reports of rice-expressed CTBs (Nochi et al. 2007; Yuki and Kiyono 2009; Tokuhara et al. 2010; Yuki et al. 2013). Systemic LTB- and CTB-specific IgG levels were similar in orally immunized mice (Figure 6b). However, production of low levels of CTB-specific IgG in orally immunized mice with maize-derived CTB mixed with maize-derived LTB suggested that LTB-specific IgG cross-reacted with CTB IgG, while CTB-specific IgG did not (Karaman et al. 2012). The level of LTB-specific mucosal IgA was increased compared to that of CTB-specific IgA. Moreover, feeding of maize-derived LTB resulted in significantly higher serum levels of LTB-specific IgA, suggesting that the maize-synthesized LTB was stable in maize tissues and was likely released slowly during digestion by LTB-immunized mice (Chikwamba et al. 2002).

Overall, these results suggest that the induction of systemic and mucosal immunity is enhanced by the combined administration of LTB and CTB, which strengthens the immune response in mice. CTB and LTBs expressed in corn (Tacket et al. 2004), maize (Karaman et al. 2012) and rice seeds (Nochi et al. 2007; Yuki et al. 2013) alone or in combination have been used to evaluate the use of corn as an edible agent against cholera and traveler’s diarrhea. The synergistic action of CTB and LTB in seeds could be enhanced by further breeding to advanced generations. In this study, we transformed rice capable of synthesizing detectable amounts of fused CTB and LTB subunit protein. This rice-based vaccine can be used as a combined vaccination system effective against both CT-based cholera and LT-based traveler’s diarrhea. The immunogenic and adjuvant abilities of the LTB and CTB proteins using mucosal immunization should be further evaluated in animal studies.

## Conclusions

In this study, we transformed rice capable of synthesizing detectable amounts of CTB and LTB subunit proteins. This can be used as a combined vaccine effective against both CT-mediated cholera and LT-mediated traveler’s diarrhea. The immunogenic and adjuvant abilities of the LTB and CTB proteins using mucosal immunization should be further evaluated in animal studies.

## Materials and methods

### Reagents and apparatus

Murashige and Skoog (MS) medium, spectinomycin, and sucrose were purchased from MB cell (CA, U.S.A). Tetracycline, cefotaxime and phosphinothricin were purchased from Duchefa (Haarlem, Netherlands). The pMJ103 vector was constructed by GreenGene BioTech (Yongin, Korea) and the plasmids (MYO51, MYO53) containing *LTB* and/or *CTB* were kindly donated from Dr. M.S. Yang (Chonbuk National University). T4 DNA ligase and restriction endonucleases were obtained from Roche (Basel, Switzerland). Gateway BP and LR recombinase systems were purchased from Invitrogen (Breda, Netherlands). A mini trans-blot cell and Mini-Protean III cell were purchased from Bio-Rad (Hercules, CA, USA). Reagents for SDS-polyacrylamide electrophoresis, such as acrylamide, bis-acrylamide, ammonium persulfate, TEMED, prestained molecular weight markers and Coomassie Brilliant Blue R-250 solution were obtained from Bio-Rad (Hercules, CA, USA). Anti-LTB polyclonal antibody was obtained from Abcam (Cambridge, MA, USA). G_M1_-ganglioside and anti-rabbit IgG-conjugated alkaline phosphatase were purchased from Santa-Cruz Biotechnology (CA, USA). Thiamine-HCl, myo-inositol, anti-CTB polyclonal antibody, and other reagents such as salts and buffer components were of analytical grade and purchased from Sigma (St. Louis, MO, USA).

### Vector construction

Two PCR fragments encoding the codon-optimized *LTB* gene (GenBank accession number: DI119695, 396 bp) and codon-optimized *CTB* gene (GenBank accession number: DI120788, 396 bp) were cloned into the pSB11 vector. Subsequently, using the Gateway BP and LR recombinase reactions, *LTB* (396 bp) and *CTB* (396 bp) fragments were cloned into the pMJ103 destination vector, including a globulin promoter (*Glb*), two recombination sites (*attB1* and *attB2*), and a potato protease inhibitor II terminator (*Pin*II). These constructed over-expression binary vectors were named pMJ103-LTB-CTB. To confirm the insertion of the *LTB* and/or *CTB* genes in pMJ103-LTB-CTB, the overexpression binary vector was digested by *Not* I (30 units/μl, Takara) for 1 h at 37°C. The restriction-digested products were analyzed by 1% agarose gel electrophoresis. Correct transfer of the *LTB* and *CTB* fragments and the presence of *Glb* and *Pin* II border regions in each vector were confirmed by DNA sequencing.

### Rice transformation

The *Agrobacterium*-mediated transformation of rice (*Oryza sativa* cv. Ilmi) was performed as described previously (Dong et al. 1996) with minor modifications. Briefly, embryogenic callus formation was induced by placing the mature embryos (scutellum side up) in 2 N6 [Chu N6 medium (MB cell, CA, U.S.A) supplemented with 2 mg L^−1^ 2,4-D, 30 g L^−1^ sucrose, 0.5 g L^−1^ proline, 0.5 g L^−1^ glutamine, 0.3 g L^−1^ casamino acids, and solidified with 2.5 g L^−1^ Phytagel (Sigma, MO, U.S.A)] at pH 5.8. After 4 weeks of incubation in the dark at 27°C, the scutellar-derived callus was used for rice callus transformation. Calli were inoculated with an overnight culture of LBA4404 harboring pMJ103-LTB-CTB and were grown to an OD_600_ of 1.5–2.0 in AAM medium supplemented with 100 μM acetosyringone (Hiei et al. 1994). Co-cultivation was performed in the dark at 24°C for 7 days in half-strength Chu N6 medium supplemented with 10 g L^−1^ glucose, 100 μM acetosyringone, 2 mg L^−1^ 2, 4-dichlorophenoxyacetic acid, and 3 g L^−1^ Phytagel at pH 5.2. After 5 weeks of incubation in the dark at 27°C, somatic embryogenic calli were transferred to regeneration medium [MS (MBcell) supplemented with 5 mg L^−1^ kinetin, 1 mg L^−1^*α*-naphthalene acetic acid, 30 g L^−1^ sorbitol, 20 g L^−1^ maltose, 3 mg L^−1^ L-phosphinothricin, 250 mg L^−1^ cefotaxime, and 4 g L^−1^ Phytagel at pH 5.8. The developing plantlets were subsequently transferred to magenta boxes and stored in pots covered with polyethylene bags for 7–14 days, after which they were transferred to a greenhouse. PCR was performed to check the presence of each fragment (*LTB* and *CTB*) in the transformed *Agrobacterium* LBA4404 compared with the control.

### DNA extraction

Genomic DNA was extracted from fresh leaf tissues of putative transgenic plants using the DNA extraction kit (Bioneer, Korea) and purified after the RNase treatment, phenol-chloroform extraction, and ethanol precipitation. Purified DNA was analyzed using 0.8% agarose gel electrophoresis and quantified using the Nano-Drop 2000 (Thermo Scientific, U.S.A). Only high-quality genomic DNA was used for PCR and Southern blot analysis.

### PCR analysis

To confirm the presence of the *LTB* and *CTB* genes in the plantlets, PCR analysis of the putative transformants was performed using the forward (LTB-F) and reverse primers (LTB-R) for the *LTB* gene, and forward (CTB-F) and reverse primers (CTB-R) for the *CTB* gene. For the *bar* gene, forward (Bar-F) and reverse primers (Bar-R) were also used (Table 2). PCR was performed in an HP thermocycler under the following conditions: 95°C for 30s, 54°C for 30s, and 72°C for 2 min for 35 cycles. PCR products were separated on a 1% agarose gel and photographed.

**Table 2:** **Primer sets used to confirm the presence of genes**

**Name**	**Sequences (5′-3′)**
Bar-F	ACTACATCGAGACAAGCACGGT
Bar-R	GCTCTACACCCACCTGCTGAAGT
CTB-F	ATGGTGAAGCTCAAGTTCGG
CTB-R	CTACTTTCCAAGTTGAGGTTC
LTB-F	ATGGTGAAGGTGAAGTGCTAC
LTB-R	ATCACATACCTCACCGAGAC
bar-F	GCATGACGTTATTTATGAGATGGGTTTT
bar-R	TGCGCGCTATATTTTGTTTTCTATCG
CT-F	GTGTTCTTCACAGTTCTCCTCTCC
CT-R	TCGTTGAGAGTATGAATTTGTGTGTTG
LT-F	CCTCACCGAGACCAAGATTGATAAG
LT-R	GTACCTCATAGCTCATCTTTCTCAGAG
Tnos-F	TCCCGTCCGCTGGTG
Tnos-R	TGCGCGCTATATTTTGTTTTCTATCG
Actin-F	CAGCCCCTCGTCTGCGATAA
Actin-R	GCCGACGTAGGCGTCCTTCT

### Trait LL lateral flow test

Three rice seeds (wild-type and transgenic seeds) were ground and homogenized in 500 μl of ddH_2_O. After 5 min of decantation, the clear supernatant was immediately used in the lateral-flow assay.

### Southern blot

Genomic DNA from the transgenic plants was digested with the *Xho*I restriction enzyme (2,000 U, Takara). The resulting fragments were fractionated on a 1% agarose gel, denatured, and transferred from the gel onto a Hybond H^+^ nylon membrane (Amersham Pharmacia Biotech) using the capillary transfer method (Sambrook and Russell 2006). The probe for the *LTB* gene was prepared by PCR from pMJ103-LTB-CTB. A total of 25 ng of *LTB* probe DNA were denatured at 100°C for 5 min. After cooling on ice for 5 min, denatured DNA was added to the mixture. After mixing with 5 μl of [α-^32^P] dCTP, probe DNA was incubated at 37°C for 10 min and labeled using Rediprime™ II Random Prime Labeling system (GE Healthcare). The reaction was terminated by adding 5 μl of 0.2 M Na-EDTA and the radiolabeled probe was purified using the PROBER™ Probe DNA purifying system (Intronbio, Korea). Purified labeled DNA was denatured by heating at 100°C for 5 min, stored on ice, and mixed with hybridization solution. Membranes were soaked in the pre-hybridization solution (6× SSC, 5× Denhardt’s reagent, 0.5% SDS and 100 μg/ml denatured fragmented salmon sperm DNA) at 65°C with gentle shaking. After 2 h, hybridization was performed in hybridization solution (6× SSC, 0.5% SDS and 100 μg/ml denatured fragmented salmon sperm DNA) containing the probe overnight at 65°C in a shaking incubator. After washing the membranes with the appropriate washing solutions and temperatures (2× SSC in 0.5% SDS at 25°C, 2× SSC in 0.1% SDS at 25°C, 0.1× SSC in 0.5% SDS at 37°C, 0.1× SSC in 0.5% SDS at 65°C, and 0.1× SSC), they were dried, covered in Saran wrap, and exposed to X-ray film (Kodak MS).

### RT-PCR analysis

Total RNAs were isolated from rice seeds (150 mg) using TRIzol reagent according to the manufacturer’s instructions (Invitrogen, Breda, Netherlands). The RNA integrity was assessed under UV light by visualization of the 28S- and 18S-rRNA bands on a 1.5% agarose gel containing ethidium bromide. For reverse transcription (RT)-PCR analysis, total RNA samples were treated with DNase before reverse transcription, according to the manufacturer’s instructions (Takara). RT-PCR amplification was performed in a reaction mixture containing 1 μg total RNA, 1 × One Step RNA PCR buffer, 0.8 U RNase inhibitor, 0.4 μM of each primer, 0.1 U AMV RTase XL, 0.1 U AMV-optimized Taq DNA polymerase, 5 mM dNTP, and 5 mM MgCl_2_ (Takara). The reaction mixture was heated at 50°C for 30 min and 94°C for 2 min for reverse transcription and inactivation of reverse transcriptase, respectively. The reaction mixture was amplified by 35 cycles, each consisting of 30 s at 94°C, 30 s at 60°C, and 30 s at 72°C. After termination of the last cycle, the samples were incubated at 72°C for 10 min and chilled at 4°C. Specific primers were used for the amplification of LTB (LT-F and LT-R) and CTB (CT-F and CT-R), resulting in an amplified products of 107 and 111 bp, respectively. *Actin*-1 (GenBank accession number: KC140126) served as an internal control. Specific primers were used for the amplification of *actin*-1 (actin-F and actin-R), resulting in an amplified product of 150 bp. The amplified PCR products were electrophoresed on a 1% agarose gel containing ethidium bromide and photographed.

### Flanking DNA sequencing and PCR of homozygous transgenic plants

Genomic DNA (500 ng) from the third-generation (T_3_) transgenic plants was digested with the restriction enzyme *Xho*I (2 U, Takara) and ligated with 5 U of T4 DNA ligase (Takara) in a 20-μl reaction volume. The reaction mixture containing T4 DNA ligase buffer and 50 pM of the adaptors was incubated at 37°C for 1 h. The first PCR was conducted using 0.5 pM specific primers (Ada1 and LB1; and Ada1 and RB1an), as shown in Table 3 and 1 μl of digestion/ligation product in a 20-μl reaction. PCR was performed using a PTC-200 thermal cycler (MJ research) under the following conditions: 95°C for 5 min, 20 cycles of 94°C for 30 s and 72°C for 1 min, followed by a final elongation step at 72°C for 10 min. Second PCRs were conducted with 5 μl of the first PCR product using specific primers (Ada2, and LB2; and Ada2, and RB2) with an initial denaturing step at 94°C for 5 min, 40 cycles of 94°C for 30 s, 60°C for 30 s, and 72°C for 1 min, followed by a final elongation at 72°C for 10 min. Amplified products were loaded on a 1% agarose gel and the PCR products were purified using the HiYield™ Gel/PCR DNA Extraction Kit (RBC) and sequenced by ABI3730XL using LB2 or RB2 primers.

**Table 3:** **Primers used for sequencing of flanking regions and selection of homozygous plants**

**Name**	**Sequences (5′-3′)**
Ada1	GCGTAATACGACTCACTATAGCAATTAACC
Ada2	GACTCACTATAGCAATTAAC
LB1an	AAGCCGTCAGGAGCTCGAATTCAGTACA
LB2	CATTAAAAACGTCCGCAATG
RB1an	AAGCTTGCTGAGTGGCTCCTTCAACG
RB2	GCTTGCTGAGTGGCTCCTTC
BP1	TATCAAAGAAGGCGGCAGGAGCCACCG
BP2	TGCCGTTTCCTGAGCACGCA
BP3	TATCAAAGAAGGCGGCAGGAGCC
LP1	TCGAAGCAGCCGATGAGCTTGA
LP2	TTGGCGTTCGGCTCCTGGAT
RB1	AGATTGTCGTTTCCCGCCTTC
RP1	TGGTATCATGGAAACGCGGGG
RP2	TCTTTAGACCCGTTGGGCCGTG
RP3	GAGAGAGATGATGTGGCATCTGACAC

The third_−_generation homozygous transgenic lines derived from LCI-7 and LCI-11 plants were analyzed by PCR of genomic DNA. The PCR was conducted with 0.5 pM of each primer to amplify the wild-type allele (RP1 and LP1; and RP2 and LP2), the T-DNA insertion allele (RP3 and BP1; and RP2 and BP2), and the second T-DNA insertion allele (LP1 and RB1; and LP2 and BP3). PCR was performed using a PTC-200 thermal cycler (MJ research) with the following reaction conditions: 95°C for 5 min, 30 cycles of 94°C for 30 s, 60°C for 1 min and 72°C for 1 min, followed by a final elongation at 72°C for 10 min.

### Quantitative real-time PCR

qRT-PCR was performed in a 96-well format in the ABI 7500 Real-Time System (Applied Biosystems) with specific primers (designed using copy caller software) used to amplify a 100-bp fragment of *LTB* (LT-F and LT-R), *CTB* (CT-F and CT-R), *Bar* (bar-F and bar-R), and T*nos* (Tnos-F and Tnos-R), as shown in Table 2. qRT-PCR was performed under the following conditions: 95°C for 10 min followed by 40 cycles of 95°C for 15 s and 60°C for 60 s. Three replicates were prepared from the same genomic DNA and the copy number of each gene. Expression levels of the *CTB*, *LTB*, T*nos* and *bar* genes were normalized to that of α-tubulin as the reference gene.

### G_M1_-ganglioside binding assay

Transgenic and wild-type rice seeds (2 g) were homogenized in 5 ml of buffer containing 0.1 M mM Tris–HCl (pH 8.0), 1 mM EDTA, 1 mM DTT, and 1× protease inhibitor cocktail (Sigma). Insoluble material was removed by centrifugation. Protein concentrations were determined using the Bradford protein assay reagent kit (Bio-Rad, Hercules, CA). The G_M1_-ganglioside binding capability of recombinant LTB and CTB proteins expressed in transgenic rice seeds was estimated based on ELISA. Briefly, a 96-well ELISA plate was coated with monosialoganglioside-G_M1_ (Santa Cruz Biotechnology) (3.0 μg ml^−1^ in sodium bicarbonate buffer) or BSA (3.0 μg ml^−1^ in sodium bicarbonate buffer) as a negative control, and incubated at 4°C overnight. Microwell strips were washed three times with approximately 200 μl of wash buffer per well. Wells were then blocked with 4% non-fat milk in PBS, washed three times with PBST and incubated with recombinant LTB (6.3 to 100 ng), authentic CTB (6.3 to 100 ng), or soluble protein from transgenic or untransformed rice seeds (4 μg) in PBS for 2 h at 37°C. Each well was then washed with PBS three times. Antisera against LTB (1:5,000 dilution) or CTB (1:5,000 dilution) in PBS buffer were added to each well and incubated for 2 h at 37°C, after which each well was washed with PBS three times. Goat anti-rabbit IgG polyclonal antiserum conjugated with AP (1:1,000 dilutions) in PBS buffer was then added to each well and incubated for 2 h at 37°C. After washing, 100 μl of *p*-nitrophenylacetate were added and the plate was incubated for 15 min. The enzyme reaction was stopped by quickly pipetting 50 μl of 2 N H_2_SO_4_ into each well. The absorbance at 405 nm was read using a microplate reader.

### Western blot analysis

Transgenic rice seeds (2 g) were homogenized in 5 ml of extraction buffer containing 100 mM Tris–HCl (pH 8.0), 1 mM DTT, 1 mM EDTA, and 0.6 mM PMSF. Insoluble material was removed by centrifugation at 12,000 rpm for 20 min. Protein concentrations were determined using the Bradford Protein Assay Reagent kit (Bio-Rad) (Bradford 1976). Total soluble proteins (100 μg; the LTB protein accounted for 0.3 μg μg^−1^ of total soluble protein and CTB accounted for 2.1 μg μg^−1^ of total soluble protein) were mixed with sample buffer (10% glycerol, 60 mM Tris–HCl pH 6.8, 2% SDS, 0.01% bromophenol blue) at room temperature and were separated using 12% non-reducing SDS-polyacrylamide gel electrophoresis and then electrophoretically transferred to a PVDF membrane (iBlot™, Invitrogen) at a constant voltage of 150 V at room temperature for 40 min in a Bio-Rad mini trans-blot cell.

The electroblotting buffer was 10 mM CAPS-10% methanol. The membrane was blocked to prevent nonspecific antibody binding using 5% non-fat milk powder in Tween/Tris-buffered saline, and probed with a rabbit polyclonal antibody against LTB or CTB at 1:10,000 dilutions for 2 h at 37°C. After washing three times, the blot was probed with HRP-conjugated secondary antibodies (1:5,000) for 2 h. The ECL chemiluminescence reagent was added to the membrane as per the manufacturer’s instructions. The membrane was exposed to Chemi-Doc detection reagent (Bio-Rad) for 1 min before being developed and fixed.

### Immunization of mice with transgenic rice seed extract

To investigate the immunogenicity of transgenic rice seeds (LCI-11), ten females BALB/c (5 weeks old) mice were administered with transgenic rice seed samples expressing both LTB and CTB. For the intraperitoneal immunization test, wild-type and transgenic rice seeds were ground and extracted in PBS. After quantifying soluble protein, test samples (100 μg in 100 μl PBS) were intraperitoneally injected into ten mice on day 1, followed by four booster injections on days 8, 15, 22, and 29. As positive controls, purified LTB (10 μg/mouse) and purified CTB (30 μg/mouse) expressed in *E. coli* as previous our report (Sim et al. 2009). To express CTB in *E. coli*, codon-optimized CTB gene was cloned at the N-terminus at Nde I site and at the C-terminus at Xho I (pET21a-CTB). The mice were divided into five groups (group 1, PBS; group 2, purified LTB; group 3, purified CTB; group 4, proteins from wild-type rice; group 5, proteins from transgenic rice) and were bled retro-orbitally every week until 5 weeks post-immunization. For oral immunization, wild-type and transgenic rice seeds were ground and suspended, and rice samples (100-mg rice powder/100-μl PBS) were orally ingested by mice on day 1 followed by eight further oral administrations on days 4, 7, 10, 13, 16, 19, 22, and 25. The amount of LTB and CTB in rice seed powders (100 mg) were approximately 90 and 110 μg, respectively. As positive controls, purified LTB (50 μg/mouse) expressed in *E. coli* was used. The mice were divided into four groups (group 1, PBS; group 2, purified LTB; group 3, wild-type rice seeds; group 4, transgenic rice seeds), and blood was sampled on day 28. Mice sera and feces were prepared, and the presence of specific antibodies to LTB and CTB was determined by ELISA. Briefly, a 96-well ELISA plate was coated with purified LTB or CTB (200 ng/well) in ELISA coating buffer (50 mM sodium bicarbonate-sodium carbonate, pH 9.6). The plate was incubated at 4°C overnight and washed with 200 μl of PBST (PBS-0.1% Tween-20) four times. The wells were then blocked with 4% non-fat milk in PBS at 37°C for 2 h. After washing the wells, diluted anti-sera (1:200, 1:1,000, 1:2,000, 1:4,000, 1: 10,000, 1:20,000, 1: 1,000,000 for intraperitoneal immunization or 1:100, 1:1,000, 1:10,000, 1: 100,000, 1: 1,000,000 for oral immunization, respectively) and diluted feces (1:100, 1:1,000, 1:10,000, 1: 100,000, 1: 1,000,000) were added to the wells and incubated at 37°C for 2 h. After washing, anti-mouse IgG-HRP (1:10,000 in both intraperitoneal and oral immunization tests) and anti-mouse IgA-HRP (1:10,000 in only oral immunization tests) were added to each well and incubated at 25°C for 1 h. After washing, 100 μl of TMB (3′, 3′, 5′, 5′-tetramethylbenzidine) containing H_2_O_2_ solution were added and the plate was incubated for 15 min at 37°C. The enzyme reaction was stopped by quickly pipetting 50 μl of 2 N H_2_SO_4_ into each well. The absorbance at 450 nm was read using a microplate reader.

Western blot analysis was performed for the detection of LTB- and CTB-specific IgG and IgA in mice sera after oral administration of LCI-11, respectively. Purified LTB or CTB proteins (50 ng) were loaded to 12% SDS-polyacrylamide gels with BSA (50 ng) as a negative control and then transferred to PVDF membranes. After blocking, membranes were probed with anti-sera (1:500) collected from mice immunized orally with LCI-11 at 4°C overnight, respectively. After washing, the blots were probed with HRP-conjugated secondary antibodies (1:1,000) for 2 h at 37°C. Membranes were developed by using the ECL reagent as the manufacturer’s protocol.
